# *Salmonella* Infantis Delays the Death of Infected Epithelial Cells to Aggravate Bacterial Load by Intermittent Phosphorylation of Akt With *SopB*

**DOI:** 10.3389/fimmu.2021.757909

**Published:** 2021-11-05

**Authors:** Bing-Xin Chu, Ya-Nan Li, Ning- Liu, Lan-Xin Yuan, Shi-Yan Chen, Yao-Hong Zhu, Jiu-Feng Wang

**Affiliations:** Department of Veterinary Clinical Sciences, College of Veterinary Medicine, China Agricultural University, Beijing, China

**Keywords:** *Salmonella* Infantis, Akt, *SopB*, apoptosis, pyroptosis, inflammation, host-pathogen interactions

## Abstract

*Salmonella* Infantis has emerged as a major clinical pathogen causing gastroenteritis worldwide in recent years. As an intracellular pathogen, *Salmonella* has evolved to manipulate and benefit from the cell death signaling pathway. In this study, we discovered that *S*. Infantis inhibited apoptosis of infected Caco-2 cells by phosphorylating Akt. Notably, Akt phosphorylation was observed in a discontinuous manner: immediately 0.5 h after the invasion, then before peak cytosolic replication. Single-cell analysis revealed that the second phase was only induced by cytosolic hyper-replicating bacteria at 3–4 hpi. Next, Akt-mediated apoptosis inhibition was found to be initiated by *Salmonella SopB.* Furthermore, Akt phosphorylation increased mitochondrial localization of Bcl-2 to prevent Bax oligomerization on the mitochondrial membrane, maintaining the mitochondrial network homeostasis to resist apoptosis. In addition, *S*. Infantis induced pyroptosis, as evidenced by increased caspase-1 (p10) and GSDMS-N levels. In contrast, cells infected with the Δ*SopB* strain displayed faster but less severe pyroptosis and had less bacterial load. The results indicated that *S*. Infantis *SopB*–mediated Akt phosphorylation delayed pyroptosis, but aggravated its severity. The wild-type strain also caused more severe diarrhea and intestinal inflammatory damage than the Δ*SopB* strain in mice. These findings revealed that *S*. Infantis delayed the cells’ death by intermittent activation of Akt, allowing sufficient time for replication, thereby causing more severe inflammation.

## Introduction

For decades, non-typhoidal salmonella (NTS) has been one of the most common foodborne zoonosis pathogens worldwide that cause host gastroenteritis. There are more than 2,600 known *Salmonella enterica* serovars, with *Salmonella enterica* serovar Infantis (*S*. Infantis) the third most prevalent serovar of human NTS infections in Europe (1, 2). It is mainly transmitted through contaminated food, such as broiler chicken and pork (3–5). Worryingly, *S*. Infantis infection has been frequently reported in many countries recently, indicating that *S*. Infantis is an emerging pathogen causing gastroenteritis worldwide (6, 7).

*Salmonella* is a Gram-negative facultative intracellular pathogen that possesses two functionally distinct T3SSs (T3SS1 and T3SS2) encoded in *Salmonella* pathogenicity islands 1 and 2 (SPI1 and SPI2), respectively (8). In epithelial cells, approximately 10–30% of *Salmonella* can escape from the *Salmonella*-containing vacuole (SCV) to the cytoplasm after internalization and replicate there (9). Cytosolic *Salmonella* proliferates faster than SCV bacteria, a phenomenon known as hyper-replication (defined as >20 bacteria/cell) (10, 11). Hyper-replicating *Salmonella* proliferates geometrically within several hours in host cells, causing cell death and extrusion and releasing invasive bacteria into the gastrointestinal tract (11).

*Salmonella* appears to have evolved to benefit from host cell signaling pathways involved in regulating cell proliferation and death (12–14). Apoptosis is a highly conserved and gene-regulated physiological programmed cell death mechanism. An increasing body of evidence indicated that the pathogenic mechanism of bacteria involves the regulation of apoptosis. The manipulation of apoptosis by *Salmonella* depends on the type of host cell and the stage of infection. Multiple apoptotic pathways are found to be rapidly activated during *Salmonella* infection of macrophages (15–17). In contrast, the apoptosis of infected epithelial cells is inhibited by *Salmonella* (18–20). It is beneficial for *Salmonella* to prolong the lifespan of infected cells, enabling bacteria to gain sufficient time for intracellular replication. *Salmonella* then induces the assembly of inflammasomes when the intracellular bacterial load increases (11, 13). Caspase-1 is subsequently activated, which converts gasdermin D (GSDMD) and the precursors of IL-1β and IL-18 to their active forms. The N-terminal fragment of GSDMD accumulates on the cell membrane, forming a polymeric pore and inducing pyroptosis, which results in the release of the intracellular bacteria and inflammatory cytokines, all of which contribute to inflammation (21–23). During an enteric infection, the induction of inflammation may be conducive to the spread of *Salmonella* in the gastrointestinal tract through the induction of rapid inflammatory pyroptosis. *Salmonella* can effectively escape from infected host cells, infect adjacent normal cells, and eliminate host immunocytes, leading to a weakened immune response (24).

In the battle between the host and *Salmonella*, two pivotal biological processes that occur are apoptosis and pyroptosis. For *Salmonella*, the regulation of cell death is also dependent on the serotype. Interestingly, *Salmonella* Typhi can replicate in macrophages without inducing cytotoxicity, while *Salmonella* Typhimurium causes severe cytotoxicity in macrophages (25). Most studies have focused on the interaction between *S*. Typhimurium and macrophages or other phagocytes, and little is known about the role of programmed cell death in controlling the pathogenesis of *S*. Infantis in epithelial cells. Furthermore, intestinal epithelial cells represent the first point of contact for *Salmonella* with the host after invasion (20, 26). In addition, there are significant differences in SPI-1 expression between *S*. Infantis and *S*. Typhimurium (27). In this study, we revealed that cytosolic *S*. Infantis phosphorylated Akt in a discontinuous manner through *SopB* to delay apoptosis and pyroptosis in infected Caco-2 cells. *S*. Infantis gained sufficient time to proliferate by prolonging the lifespan of infected cells, eventually causing pyroptosis, which was accompanied by the release of inflammatory factors and bacteria. This created favorable conditions for the spread and infection of *S*. Infantis.

## Materials and Methods

### Reagents and Antibodies

The reagents and antibodies used in the study are shown in **Table 1**.

**Table 1:** Reagents and antibodies.

Reagents and antibodies	Catalog number	Company/Brand	Origin
Mito-Tracker Red CMXRos	C1035	Beyotime Biotechnology	Shanghai, China
Hoechst 33342	C1025	Beyotime Biotechnology	Shanghai, China
SC79	SF2730	Beyotime Biotechnology	Shanghai, China
MK2206	SF2712	Beyotime Biotechnology	Shanghai, China
Cell Mitochondria Isolation Kit	C3601	Beyotime Biotechnology	Shanghai, China
Enhanced Cell Counting Kit-8	C0042	Beyotime Biotechnology	Shanghai, China
Alexa Fluor 488-labeled Goat Anti-Rabbit lgG(H+L)	A0423	Beyotime Biotechnology	Shanghai, China
4’,6’-diamidino-2-phenylindole (DAPI) solution	C0060	Beijing Solarbio Science & Technology Co., Ltd.	Beijing, China
Calcein-AM/PI	CA1630	Beijing Solarbio Science & Technology Co., Ltd.	Beijing, China
Gentamycin Sulfate	G8170	Beijing Solarbio Science & Technology Co., Ltd.	Beijing, China
Carbonyl cyanide 3-chlorophenylhydrazone (CCCP)	C6700	Beijing Solarbio Science & Technology Co., Ltd.	Beijing, China
Chlorquine diphosphate salt	C6628	Sigma-Aldrich	St. Louis, USA
Triton X-100	T8787	Sigma-Aldrich	St. Louis, USA
Dulbecco’s Modified Eagle Medium (DMEM)/High Glucose	SH30022.01	GE Healthcare Life Sciences HyClone Laboratories	Utah, USA
Phosphate buffered saline (PBS)	SH30256.01	GE Healthcare Life Sciences HyClone Laboratories	Utah, USA
Fetal bovine serum (FBS)	10099141	Thermo Fisher Scientific	Rockford, USA
Annexin V-PE/7-AAD Apoptosis Detection Kit	A213	Vazyme Biotech Co., Ltd	Jiangsu, China
TUNEL FITC Apoptosis Detection Kit	A111-01	Vazyme Biotech Co., Ltd	Jiangsu, China
Rabbit Anti-LAMP 1 polyclonal antibody	21997-1-AP	Proteintech Group Inc	Rosemont, USA
Rabbit Anti-Occludin polyclonal antibody	27260-1-AP	Proteintech Group Inc	Rosemont, USA
Rabbit Anti-Claudin-1 polyclonal antibody	13050-1-AP	Proteintech Group Inc	Rosemont, USA
Rabbit Anti-Caspase 9/p35/p10 polyclonal antibody	10380-1-AP	Proteintech Group Inc	Rosemont, USA
Rabbit Anti-Bax polyclonal antibody	50599-2-Ig	Proteintech Group Inc	Rosemont, USA
Rabbit Anti-Bcl-2 polyclonal antibody	12789-1-AP	Proteintech Group Inc	Rosemont, USA
Rabbit Anti-VDAC1 polyclonal antibody	55259-1-AP	Proteintech Group Inc	Rosemont, USA
Rabbit Anti-Phospho-Caspase-9 (Ser196) polyclonal antibody	28794-1-AP	Proteintech Group Inc	Rosemont, USA
Rabbit Anti-Cytochrome c monoclonal antibody	66264-1-Ig	Proteintech Group Inc	Rosemont, USA
Mouse Anti-Beta ACTIN monoclonal antibody	60008-1-lg	Proteintech Group Inc	Rosemont, USA
HRP-conjugated Affinipure Goat Anti-Rabbit IgG(H+L)	SA00001-2	Proteintech Group Inc	Rosemont, USA
HRP-conjugated Affinipure Goat Anti-Mouse IgG(H+L)	SA00001-1	Proteintech Group Inc	Rosemont, USA
Rabbit Anti-Cleaved Caspase-3 monoclonal antibody	9661T	Cell Signaling Technology	Danvers, USA
Mouse Anti-Cleaved PARP monoclonal antibody	9548T	Cell Signaling Technology	Danvers, USA
Rabbit Anti-Phospho-Akt (Ser473) monoclonal antibody	4060T	Cell Signaling Technology	Danvers, USA
Rabbit Anti-Phospho-Akt (Thr308) monoclonal antibody	13038T	Cell Signaling Technology	Danvers, USA
Rabbit Anti- Caspase-1 monoclonal antibody	24232S	Cell Signaling Technology	Danvers, USA
Rabbit Anti-ZO-1 polyclonal antibody	40-2300	Thermo Fisher Scientific	Rockford, USA
Rabbit Anti-Phospho-Bad (Ser136) polyclonal antibody	ab15098	Abcam	Cambridge, UK
Rabbit Anti GSDMD-N monoclonal antibody	ab215203	Abcam	Cambridge, UK

### Bacterial Strains

The *S*. Infantis wild-type strain CAU1508 was isolated from the intestinal contents of diarrhea piglets. *S*. Infantis with the pFPV-mCherry plasmid has been previously described (12). The *SopB* mutant strain was derived from the parental *S*. Infantis wild-type strain CAU1508 and constructed using the λ-Red homologous recombination system.

### Host Cell Infection and Enumeration of Intracellular Bacteria

Caco-2 cells were purchased from Kunming Cell Bank of Chinese Academy of Sciences. The Caco-2 cells were cultured in DMEM/High Glucose media supplemented with 10% FBS and 1% penicillin streptomycin at 37°C in a 5% CO_2_ incubator. Cells were seeded in six-well (1×10^6^ cells per well) or 24-well culture plates (1×10^5^ cells per well) and infected when the cell density reached 60% (this is to ensure that bacteria can infect as many cells as possible). *Salmonella* was grown in LB medium overnight with shaking at 200 rpm and 37°C, then subcultured in 10 ml fresh LB medium (1:40) with shaking under the same conditions for 4 h. Following that, the bacteria were centrifuged at 4,000 g for 15 min at room temperature and resuspended in PBS. The entire infection was according to the experimental procedure of the gentamicin protection assay. The monolayers were infected at an MOI of ~50 for 15 min and washed three times with PBS supplemented with gentamicin (100 μg/ml) to remove extracellular bacteria. Cells were then incubated in fresh growth medium containing gentamicin (100 mg/ml) for 2 h, followed by growth medium supplemented with gentamicin (10 μg/ml) until the infection was complete. After 15 min of *Salmonella* invasion, the time was 0 hpi (hours post-infection), and the infection lasted for 8 h in total. For groups that required treatment with MK2206 (1 μM) and SC79 (25 μM), both drugs were added 2 h before infection, and their concentrations remained unchanged in the medium until the end of the experiment. For the positive control of apoptosis, cells treated with CCCP (Carbonyl cyanide 3-chlorophenylhydrazone, an apoptosis inducer, 50 μM) for 1 h before other experiments were carried out (immunoblotting or immunofluorescence).

### Cell Viability Assay

Cell viability was determined using the Cell Counting Kit-8. Cells were seeded in 96-well culture plates. At the end of each treatment, the medium was removed and replaced with 100 μl medium containing 10 μl fresh CCK-8 solution, then incubated at 37°C for 2 h. Following that, absorbance was measured at 450 nm. Experiments were performed six times on each group to ensure the authenticity of the results.

### Enumeration of Intracellular Bacteria

In order to quantify viable intracellular bacteria, monolayers in six-well plates were washed three times with PBS containing gentamicin (100 mg/ml) for 5 min each time before being lysed in 1 ml of 0.3% (v/v) Triton X-100. Serial dilutions were plated on LB agar plates. For quantification of intracellular cytosolic bacteria, cells were co-incubated with media containing chloroquine (700 μM) for 1 h before being solubilized in 1 ml of 1% (v/v) Triton X-100, then plated on LB agar plates.

### Apoptosis Assay

After *Salmonella* infection, Caco-2 cells were collected, washed twice with PBS, suspended in 100 μl 1× binding buffer, and stained with the Annexin V-PE/7-AAD Apoptosis Detection Kit. Next, 5 μl each of Annexin V-PE and 7-ADD were added to each sample and incubated in the dark for 15 min. Flow cytometry analysis was performed using a BD FACSVerse™ Flow Cytometer.

### Calcein-AM/PI Assay

Cells were seeded into 24-well plates. After 8 h of *Salmonella* infection, media was removed and the cells were washed with PBS. Following that, 2 ml of 1× assay buffer supplemented with Calcein-AM (2 μM) and PI (4.5 μM) was added to the cells and incubated at 37°C for 15 min. The samples were then examined with a fluorescence microscope under a 490 ± 10 nm excitation filter.

### Western Blotting

Total protein of Caco-2 cells was extracted using RIPA buffer (Solarbio, Beijing, China) containing a protease/phosphatase inhibitor cocktail (Cell Signaling Technology, USA) on ice for 30 min. Protein concentration was quantified using the BCA Protein Assay kit (23227, ThermoFisher Scientific). SDS-PAGE was used to separate protein samples, which were then transferred to polyvinylidene fluoride membranes. After incubation with 5% skim milk, the membranes were incubated with primary antibodies. Further details about the primary antibodies are described in the **Table 2**. Next, the membranes were incubated with secondary antibodies, then coated with ECL immunoblotting substrate. Images were captured using a Tanon 6200 chemiluminescence imaging workstation.

**Table 2:** The dilution ratio of primary antibodies.

Antibody	Dilution rate
Rabbit Anti-LAMP 1 polyclonal antibody	1:2,000
Rabbit Anti-Occludin polyclonal antibody	1:1,500
Rabbit Anti-Claudin-1 polyclonal antibody	1:2,000
Rabbit Anti-Caspase 9/p35/p10 polyclonal antibody	1:500
Rabbit Anti-Bax polyclonal antibody	1:5,000
Rabbit Anti-Bcl-2 polyclonal antibody	1:1,000
Rabbit Anti-VDAC1 polyclonal antibody	1:1,000
Rabbit Anti-Phospho-Caspase-9 (Ser196) polyclonal antibody	1:1,000
Rabbit Anti-Cytochrome c monoclonal antibody	1:5,000
Mouse Anti-Beta ACTIN monoclonal antibody	1:5,000
Rabbit Anti-Cleaved Caspase-3 monoclonal antibody	1:1,000
Mouse Anti-Cleaved PARP monoclonal antibody	1:1,000
Rabbit Anti-Phospho-Akt (Ser473) monoclonal antibody	1:2,000
Rabbit Anti-Phospho-Akt (Thr308) monoclonal antibody	1:2,000
Rabbit Anti- Caspase-1 monoclonal antibody	1:1,000
Rabbit Anti-ZO-1 polyclonal antibody	1:500
Rabbit Anti-Phospho-Bad (Ser136) polyclonal antibody	1:500
Rabbit Anti GSDMD-N monoclonal antibody	1:1,000

### Immunofluorescence

Cells were fixed with 4% paraformaldehyde and permeabilized with 1% (v/v) Triton X-100. Following that, the samples were blocked with 2% bovine serum albumin at room temperature. The samples were then incubated with anti-LAMP1 (1:100); anti-Cleaved-caspase-3 (1:100); anti-Cytochrome c (1:50); anti-Phospho-Akt (Thr308) (1:800); anti-Tom-20 (1:200) at 4°C overnight. The Mito-Tracker Red CMXRos was used to label mitochondria. Next, the samples were incubated with secondary antibodies at room temperature. Either DAPI or Hoechst 33342 was used to stain the DNA. Images were taken using a Nikon A1 confocal laser scanning microscope.

### Mitochondrial Network Morphology Assay

Mitochondria were labeled with Tom-20 and imaged using confocal microscopy. The Mitochondrial Network Analysis (MiNA) toolset, which consists of a relatively simple pair of macros using existing ImageJ plug-ins, was used to analyze the mitochondrial networks.

### Animal Infection Experiment

All animal work was performed in accordance with the Guidelines for Laboratory Animal Use and Care of the Chinese Center for Disease Control and Prevention and the Rules for Medical Laboratory Animals (1998) of the Chinese Ministry of Health. A total of 36 six-week-old male C57BL/6 mice were obtained from Charles River Laboratory Animal Technology Co., Ltd (Beijing, China). Mice were provided food and water *ad libitum* throughout the entire experiment. All mice were administered a single dose of streptomycin (15 mg per mouse) *via* gastric gavage before being infected *via* gavage 24 h later (2 × 10^6^ *Salmonella* in 200 μl of PBS). Control mice were orally fed an equal volume of PBS. The mice were then euthanized, and their ileum tissues were harvested 3 days after infection. Mice feces were homogenized in 1 ml of PBS, and serial dilutions were plated on LB agar plates to quantify bacterial burdens.

### Assessment of Diarrhea Degree

The severity of diarrhea was evaluated using the fecal score and the dry/wet weight of fecal pellets. The fecal scoring criteria were as follows: 1 (normal stool); 2 (slightly wet, soft stool and formed stool); 3 (wet and unformed stool with mucus); 4 (watery stool). In order to determine the fecal dry/wet weight ratio, mice were separately placed in a clean cage, without food or water. Next, 0.5 g of feces was collected and weighed. Following that, the feces were placed in a 60°C oven for 24 h until the weight change was less than 1% before being weighed. The dry/wet weight ratio was then calculated.

### Histopathologic Section

In order to evaluate ileal pathology, the mid-segments of the ileum were excised, rinsed with saline, then fixed with 4% paraformaldehyde for 48 h. Paraffin-embedded tissue samples were sectioned (3 μm) and stained with hematoxylin and eosin. A neutral resin was used for sealing. Tissue sections were observed and imaged using an Olympus CX23 microscope equipped with an imaging system.

### Real-Time Quantitative PCR

For gene expression analysis, total RNA was extracted from ileal tissues using the Trizol reagent (Invitrogen, Carlsbad, CA, USA). RNA transcription was performed using the PrimeScriptTM RT Reagent Kit according to the manufacturer’s instructions (RR047A, TaKaRa, Japan). Quantitative real-time RT-PCR was performed using the SYBR Green PCR Master Mix (LS2062, Promega, USA). The cycle threshold (CT) values of target genes were normalized to the CT value of hypoxanthine gene hypoxanthine phosphoribosyl-transferase. The results were presented as fold-change using the 2^−ΔΔCT^ method. Primer sequences for PCR are listed in **Table 3**.

**Table 3:** Real-time PCR primers.

Primer name	Direction^a^	Sequence (5′→3′)
TNF-α	F	CCTGTAGCCCACGTCGTAG
	R	GGGAGTAGACAAGGTACAACCC
IL-6	F	TCTATACCACTTCACAAGTCGGA
	R	GAATTGCCATTGCACAACTCTTT
IL-1β	F	GAAATGCCACCTTTTGACAGTG
	R	TGGATGCTCTCATCAGGACAG
IL-18	F	TGTTGAGCATGAAAAGCCTCTAT
	R	AGGTCTCCCGAATTGGAAAGG
IL-22	F	ATGAGTTTTTCCCTTATGGGGAC
	R	GCTGGAAGTTGGACACCTCAA
IFN-γ	F	ATGAACGCTACACACTGCATC
	R	CCATCCTTTTGCCAGTTCCTC
β-Actin	F	CTACCTCATGAAGATCCTGACC
	R	CACAGCTTCTCTTTGATGTCAC

a F, forward; R, reverse.

### Statistical Analysis

All statistical analysis was performed using GraphPad Prism 7 with a one-way ANOVA or a t-test with Bonferroni correction. Data were presented as means ± SEM. *P* < 0.05 was considered statistically significant.

## Results

### *S*. Infantis Can Hyper-Replicate in Caco-2 Cells

**Figure 1:**
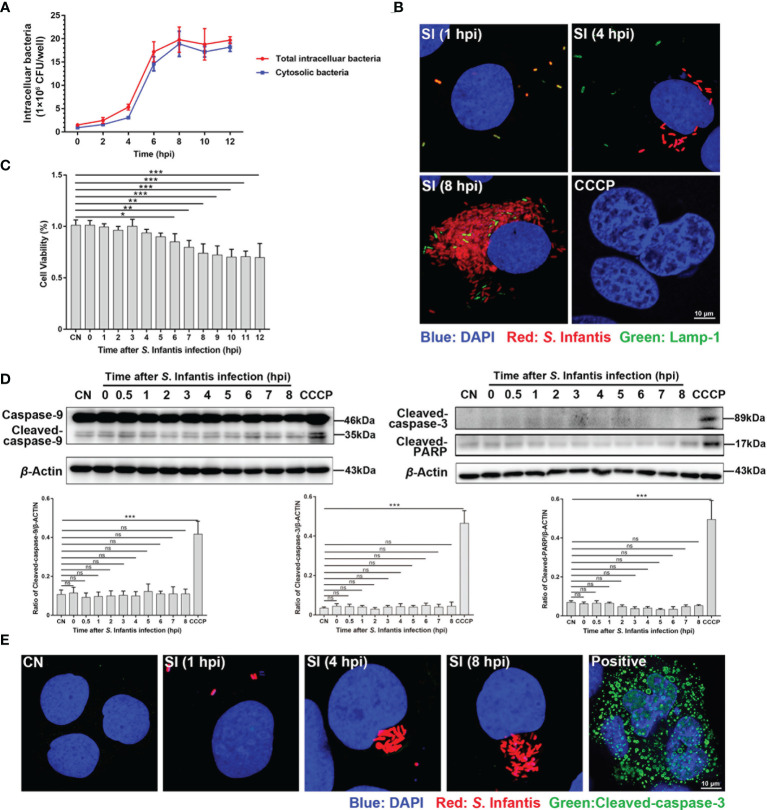
Infected cells containing hyper-replicating cytosolic *S*. Infantis did not undergo apoptosis. **(A)** Detection of intracellular bacterial load using the gentamicin protection assay, without (total intracellular bacteria, red curve) or with (cytosolic bacteria, blue curve) chloroquine. Data were obtained from three independent replicates of each sample at 0, 2, 4, 6, 8, 10, and 12 hpi. **(B)** Detection of the distribution and proportion of two subpopulations in Caco-2 cells by immunofluorescence staining. SI, *S*. Infantis. Red: *Salmonella*. Green: Lamp-1. Blue: DAPI. Scale bar, 10 µm. **(C)** Cell viability assay of Caco-2 cells infected with *S*. Infantis within 12 h. CN, control. **(D)** Western blot analysis of Caspase-9, Cleaved-caspase-3, and Cleaved-PARP protein expression levels within 12 h after bacterial infection. A total of 10 time points were set for sampling: 0, 0.5, 1, 2, 3, 4, 5, 6, 7, 8 hpi. CN, control. ns, no significant difference. **(E)** Immunofluorescence staining analysis of Cleaved-caspase-3 in Caco-2 cells. Samples were treated at 1, 4, and 8 hpi, respectively. CN, control; SI, *S*. Infantis. Red: *S*. Infantis. Green: Cleaved-caspase-3. Blue: DAPI. Scale bar, 10 µm. CCCP was the apoptosis-positive control group. Data were presented as the mean ± SEM from three independent experiments (n = 3). *P < 0.05, **P < 0.01, ***P < 0.001.

The curves showed that the bacterial load began to rapidly increase at 4 hpi and peaked at 8 hpi (**Figure 1A**). We discovered two bacterial subpopulations in the cell by adding chloroquine (used to selectively kill vacuolar bacteria): one in the SCV and the other free in the cytoplasm. The bacterial load was mainly contributed by cytosolic bacteria (**Figure 1A**). Next, we used mCherry-*S*. Infantis to infect the cells and LAMP-1 to label SCV for confirmation. Confocal microscopy images revealed that cytosolic *S*. Infantis was the absolute dominant subpopulation (**Figure 1B**).

### *S*. Infantis Infection Does Not Induce Apoptosis of Caco-2 Cells

The cell survival rate significantly decreased at 6 hpi and remained almost unchanged after 8 hpi (**Figure 1C**). Therefore, we focused on the 0–8 hpi period, which encompasses the peak of cytosolic replication and the low phase of cell viability. The results showed that Caspase-9, Cleaved-caspase-3, and Cleaved-PARP were not activated during *S*. Infantis infection (**Figure 1D**). Morphological analysis of apoptosis revealed that *S*. Infantis infection did not result in apoptotic characteristics of the nucleus (**Figures 1B, E**). These findings indicated that cytosolic *S*. Infantis hyper-replicated in Caco-2 cells without inducing apoptosis.

### Cytosolic *S*. Infantis Inhibits Apoptosis by Intermittently Phosphorylating Akt

**Figure 2:**
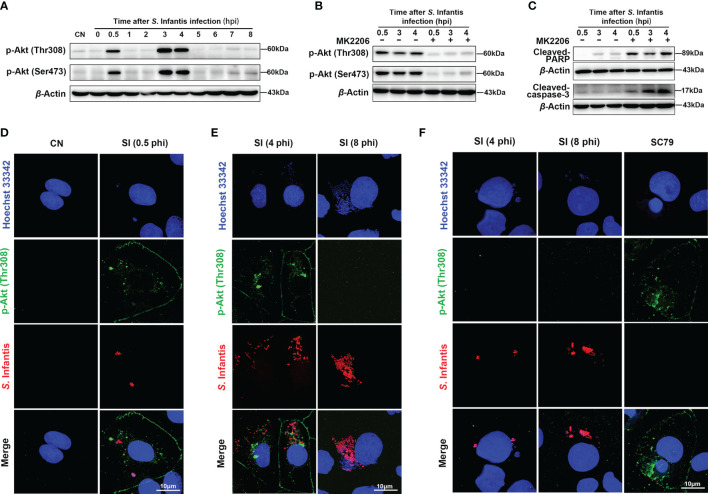
Cytosolic hyper-replicating *S*. Infantis inhibited apoptosis by intermittently phosphorylating Akt. **(A)** Western blot analysis of p-Akt (Ser473) and p-Akt (Thr308) protein expression levels at 0, 0.5, 1, 2, 3, 4, 5, 6, 7, 8 hpi, respectively. CN, control. **(B)** Immunoblotting verified the inhibitory effect of MK2206 on p-Akt (Ser473) and p-Akt (Thr308) protein expression levels at 0.5, 3, and 4 hpi. **(C)** Western blot analysis of Cleaved-caspase-3 and Cleaved-PARP protein expression levels at 0.5, 3, and 4 hpi after after p-Akt was suppressed by MK2206. **(D–F)** Immunofluorescence staining analysis p-Akt (Thr308) distribution. CN, control; SI, *S*. Infantis. **(D)** Distribution of p-Akt (Thr308) in all infected cells at 0.5 hpi. **(E)** Expression of p-Akt (Thr308) in infected cells containing cytosolic hyper-replicating *S*. Infantis at 4 and 8 hpi. **(F)** Expression of p-Akt (Thr308) in infected cells without cytosolic hyper-replicating *S*. Infantis at 4 and 8 hpi. At least 100 cells were counted for each group. Red: *S*. Infantis. Green: p-Akt (Thr308). Blue: Hoechst 333342. Data were presented as the mean ± SEM from three independent experiments (n = 3).

Akt regulates cell survival and suppresses apoptosis *via* phosphorylation at Thr308 and Ser473 sites (27–29). Several studies have found that Akt is constantly phosphorylated after *S*. Typhimurium invades epithelial cells (18, 20). In this study, we hypothesized that continuous Akt phosphorylation contributes to the inhibition of apoptosis. As seen in **Figure 2A**, phosphorylation of Akt occurred in a discontinuous manner during *S*. Infantis infection. The first phase occurred at 0.5 hpi and rapidly decreased to near the background level. The second phase was observed at 3–4 hpi, with the expression of p-Akt higher than in the first phase. The levels of Cleaved-caspase-3 and Cleaved-PARP significantly increased after Akt phosphorylation was inhibited by MK2206, an Akt inhibitor that inhibits Akt phosphorylation at Thr 308 and Ser 473 (**Figures 2B, C**).

Next, we explored which parts of the bacteria induced Akt phosphorylation. At 0.5 hpi, Akt phosphorylation was observed in all infected cells (**Figure 2D**). Images at 4 hpi revealed high levels of p-Akt in cells containing cytosolic hyper-replicating *S*. Infantis (bacteria number >20), which were not observed at 8 hpi (**Figure 2E**). In infected cells without cytosolic bacteria, phosphorylation of p-Akt was not detected at 4 and 8 hpi (**Figure 2F**). These results demonstrated that *S*. Infantis-induced Akt phosphorylation occurred in two distinct phases. The first phase is widely induced after the invasion and rapidly depleted within 30 min, whereas the second phase is only induced by cytosolic bacteria at 3–4 hpi. Both phases of Akt phosphorylation inhibited apoptosis of the infected cells.

### Inhibition of Apoptosis by Akt Intermittent Phosphorylation Is Mediated by Cytosolic *S*. Infantis *SopB*

**Figure 3:**
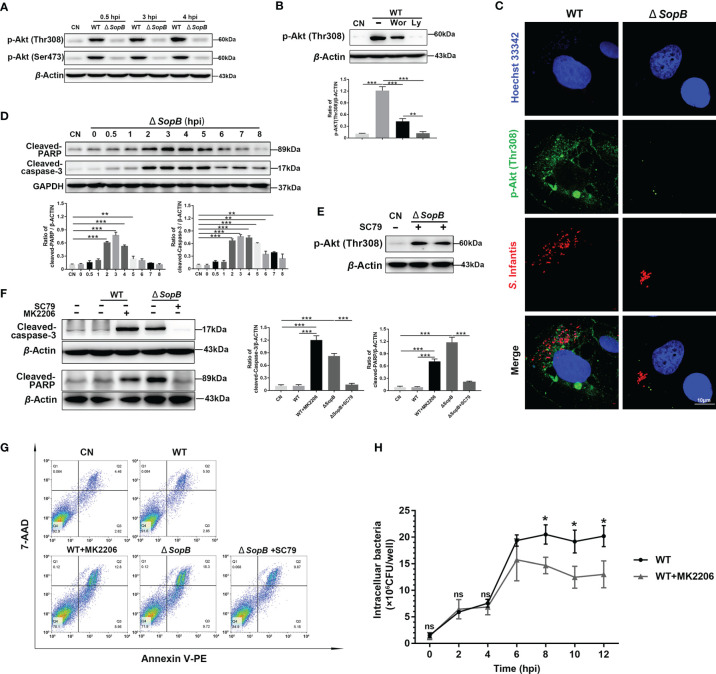
Inhibition of apoptosis by intermittent Akt phosphorylation was mediated by Cytosolic *S*. Infantis *SopB*. **(A)** Western blot analysis of p-Akt (Ser473) and p-Akt (Thr308) protein expression levels after WT *S*. Infantis or *SopB* mutant infection at 0.5, 3, and 4 hpi. CN, control. **(B)** Immunoblotting verified the repressive effects of Ly294002 (Ly) and Wortmannin (Wor) on p-Akt (Thr308) protein expression levels after infection with WT *S*. Infantis. CN, control. **(C)** Immunofluorescence staining of p-Akt (Thr308) after infection with WT *S*. Infantis or the *SopB* mutant at 4 hpi. Red: *S*. Infantis. Green: p-Akt (Thr308). Blue: Hoechst 333342. **(D)** Western blot analysis of Cleaved-caspase-3 and Cleaved-PARP protein expression levels within 8 h after *SopB* mutant infection. CN, control. **(E)** Immunoblotting verified the activation of SC79 on p-Akt (Thr308) protein expression level after *SopB* mutant infection at 4 hpi. CN, control. **(F, G)** Apoptosis was evaluated after infection with WT *S*. Infantis or the *SopB* mutant at 4 hpi. In the process of bacterial infection, the phosphorylation level of Akt was regulated by the addition of MK2206 or SC79. **(F)** The protein levels of Cleaved-caspase-3 and Cleaved-PARP were analyzed using Western blotting. **(G)** The proportion of apoptotic cells was detected using flow cytometry. **(H)** Detection of intracellular bacterial load using the gentamicin protection assay without (total intracellular bacteria, black curve) or with (total intracellular bacteria, gray curve) MK2206. ns, no significant difference. Data were presented as the mean ± SEM from three independent experiments (n = 3). *P < 0.05, **P < 0.01, ***P < 0.001.

Notably, the SPI1 effector *SopB*, which contributes to invasion and SCV maturation, has 4-phosphatase activity, which can induce Akt activation during *S*. Typhimurium infection (18, 20). As expected, the p-Akt level was almost completely diminished in cells infected with the *ΔSopB* mutant (**Figures 3A, C**). Interestingly, LY294002 (Ly, a pan Akt inhibitor) completely inhibited Akt phosphorylation, but there was a certain expression of p-Akt after Wortmannin (Wor, a PI3K/Akt inhibitor) treatment (**Figure 3B**). Furthermore, infection with the *SopB* mutant induced apoptosis, as demonstrated by nuclear chromatin condensation and increase of Cleaved-caspase-3 and Cleaved-PARP levels (**Figures 3C, D**). The SC79 (an Akt phosphorylation activator) was used to activate Akt (**Figure 3E**), with findings confirming that *SopB*-mediated Akt intermittent phosphorylation inhibited apoptosis of infected cells (**Figures 3F, G**). Importantly, wild-type (WT) *S*. Infantis had enough time for intracellular replication, resulting in increased bacterial load by inhibiting apoptosis (**Figure 3H**).

### *SopB* Mediated Akt Phosphorylation Inhibits Apoptosis by Maintaining Mitochondrial Dynamic Network Homeostasis

**Figure 4:**
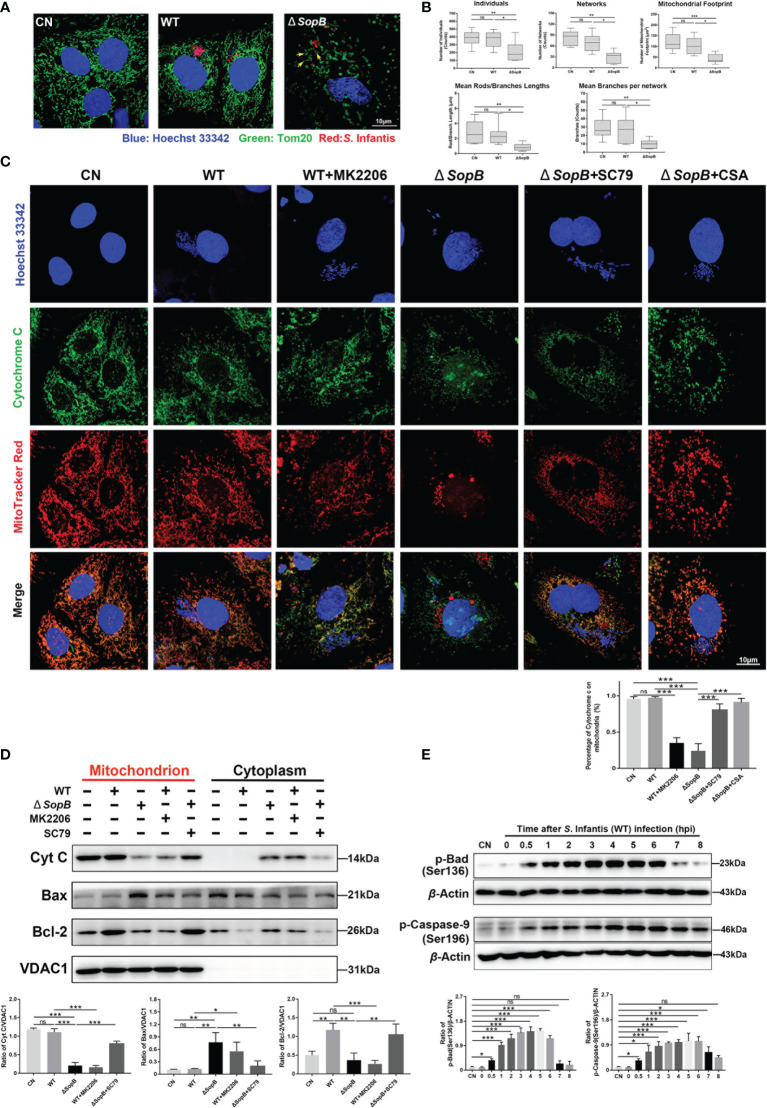
*SopB*-mediated Akt phosphorylation inhibited apoptosis by maintaining mitochondrial dynamic network homeostasis. In **(A–D)**, all cell samples were collected and processed at 4 hpi after infection with *S*. Infantis. Immunofluorescence analysis of mitochondrial network after infection with WT *S*. Infantis or the *SopB* mutant. Red: *Salmonella*; Green: Tom20 (mitochondria); Blue: Hoechst 333342. **(B)** Mitochondrial network analysis using the MiNA toolset of Image (J) **(C)** Co-localization of cytochrome c and mitochondria was detected using immunofluorescence. In the bacterial infection process, the phosphorylation level of Akt was regulated by the addition of MK2206 or SC79. CSA was used to inhibit the opening of the mitochondrial permeability transition pore (MPTP) Red: Mitochondria; Green: Cytochrome c; Blue: Hoechst 333342 (Nucleus and bacteria). **(D)** Detection of Cytochrome c, Bcl-2, and Bax protein levels in mitochondrial and cytoplasmic protein after infection with WT *S*. Infantis or the *SopB* mutant. In the bacterial infection process, the phosphorylation level of Akt was regulated by the addition of MK2206 or SC79. **(E)** Western blot analysis of p-Caspase-9 (Ser136) and p-Bad (Ser196) protein expression levels within 8 h after infection with *S*. Infantis. Data were presented as the mean ± SEM from three independent experiments (n = 3). *P < 0.05, **P < 0.01, ***P < 0.001. CN, control. ns, no significant difference.

The mitochondrion is the primary control organelle responsible for endogenous apoptosis. In normal cells, individual mitochondria connect to form tubules and shape dynamic networks through continuous division and fusion (30). Therefore, we evaluated the morphology of the infected cells’ mitochondrial network. At 4 hpi, the mitochondria of WT *S*. Infantis–infected cells still maintained an abundant network structure, but infection with the *SopB* mutant disrupted the mitochondrial network (**Figure 4A**). The mitochondrial cavity also appeared to be expanding, as evidenced by the ring-shaped structure (yellow arrows) (**Figure 4A**). Morphological analysis of the mitochondrial network revealed that infection with the WT strain had no discernible effect on the dynamics of the mitochondrial network (**Figure 4B**).

The key events of mitochondria-mediated apoptosis are the opening of mitochondrial permeability transition pore (MPTP) and the release of cytochrome c (31, 32). In the WT group, cytochrome c and mitochondria remained co-localized, while mk2206 treatment resulted in cytochrome c translocation from the mitochondria to the cytoplasm (**Figures 4C, D**). Infection with the *SopB* mutant resulted in massive cytochrome c release into cytoplasm, which was reversed by the addition of SC79, partially restoring the mitochondrial network (**Figures 4C, D**). Interestingly, co-localization of cytochrome c and mitochondria was also restored by the addition of CSA (a MPTP blocker) (**Figure 4C**). The mitochondrial membrane permeability may be affected by p-Akt induced by *SopB*, and the Bcl-2 family regulates the permeability of the mitochondrial outer membrane by inhibiting Bax translocation from the cytosol to the mitochondria (33, 34). We extracted mitochondrial protein and detected the distribution of Bcl-2 and Bax. As shown in **Figure 4D**, WT *S*. Infantis infection significantly increased the distribution of Bcl-2 in the mitochondria, while the addition of mk2206 decreased Bcl-2 and increased the distribution of Bax in the mitochondria. Infection with the *SopB* mutant also resulted in the decrease of Bcl-2 and the increase of Bax in mitochondria, which could be reversed by adding SC79. In addition, WT *S*. Infantis infection could phosphorylate Bad and Caspase-9 (**Figure 4E**), which enhanced the inhibition of apoptosis. In summary, *S*. Infantis-mediated Akt phosphorylation by *SopB* maintained mitochondrial dynamic network homeostasis, hence suppressing the apoptosis in infected cells.

### *SopB*-Mediated Akt Phosphorylation Delays Pyroptosis by Inhibiting Caspase-1

**Figure 5:**
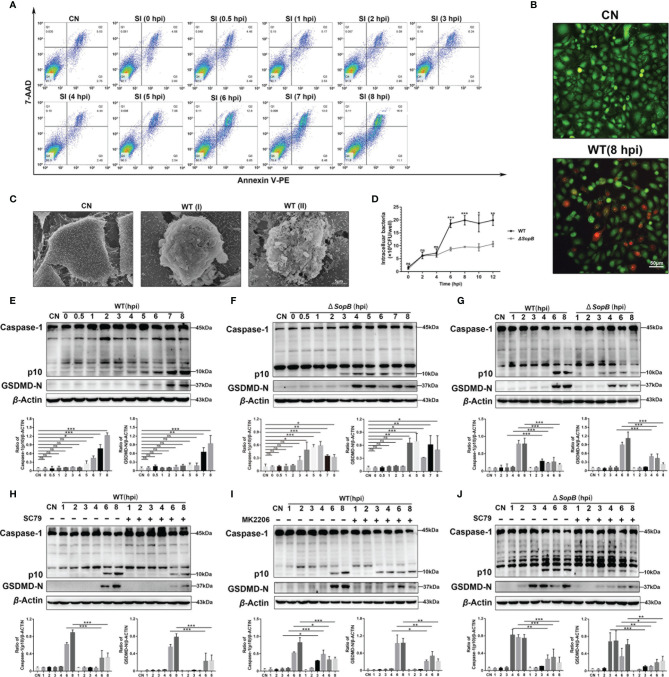
*SopB*-mediated Akt phosphorylation delayed pyroptosis. **(A)** The proportion of apoptotic cells within 8 h after infection with WT *S*. Infantis was detected using flow cytometry. **(B)** Calcein-AM/PI was used to evaluate cell membrane permeability after 8 h of infection with WT *S*. Infantis. Green: Calcein-AM; Red: PI. **(C)** Observation of the apical surface of Caco-2 cell monolayer ultrastructure using scanning electron microscopy. WT (I) (6 hpi) and WT (II) (8 hpi) showed that the WT *S*. Infantis–infected cell was bacteria-laden, which were extruded from the monolayer. **(D)** Detection of intracellular bacterial load using the gentamicin protection assay at 0, 2, 4, 6, 8, 10, and 12 hpi after infection (Black curve: WT *S*. Infantis; Gray curve: *SopB* mutant). **(E–J)** Western blot analysis of Caspase-1 and GSDMD-N protein levels after infection with WT *S*. Infantis or the *SopB* mutant within 8 h. **(E)** Infection with WT *S*. Infantis; **(F)** infection with the *SopB* mutant; **(G)** comparison of WT *S*. Infantis and the *SopB* mutant; **(H)** infection with WT *S*. Infantis in the presence of SC79; **(I)** infection with WT *S*. Infantis in the presence of MK2206; **(J)** infection with the *SopB* mutant infection in the presence of SC79. Data were presented as the mean ± SEM from three independent experiments (n = 3). *P < 0.05, **P < 0.01, ***P < 0.001. CN, control; SI, *S*. Infantis. ns, no significant difference.

Flow cytometry results showed that the proportion of 7-ADD^+^/Annexin-V PE^+^ cells significantly increased during 6–8 hpi, and the cells entered later stage of apoptosis without the early phase (**Figure 5A**). This indicated that there was a change in the membrane permeability of infected cells. In order to validate this conjecture, we performed double staining with Calcein-AM and PI during infection with *S*. Infantis. Images revealed that a subset of cells in the *S*. Infantis infection group had been damaged, as evidenced by PI-positive staining (**Figure 5B**). The scanning electron microscope images revealed that plasmalemma was destroyed and bacteria had been drilled out along the pores (**Figure 5C**), suggesting the occurrence of pyroptosis.

Next, the protein markers of pyroptosis, caspase-1 (p10), and GSDMD-N were examined. Infection with WT *S*. Infantis significantly activated pyroptosis (**Figure 5E**). Surprisingly, the *ΔSopB* strain induced caspase-1 (p10) and GSDMD-N activation 2 h earlier than WT *S*. Infantis (**Figure 5F**). However, the *SopB* mutant induced lower levels of caspase-1 (p10) and GSDMD-N compared to the WT strain, indicating a weaker degree of pyroptosis induced by the *SopB* mutant (**Figure 5G**). A recent study reported that p-Akt suppressed inflammasome activation in *Salmonella*-infected macrophages (35). Pyroptosis can be regulated by *S*. Infantis through p-Akt. MK2206 significantly reduced the levels of caspase-1 (p10) and GSDMD-N induced by WT *S*. Infantis, while SC79 treatment caused the WT strain to display a similar regulation as the *ΔSopB* strain (**Figures 5H, I**). Furthermore, SC79 treatment resulted in a significant decrease in caspase-1 (p10) and GSDMD-N levels during infection with the *ΔSopB* strain (**Figure 5J**), indicating that Akt phosphorylation both delayed pyroptosis and aggravated the severity of pyroptosis of infected Caco-2 cells. Intracellular bacterial load detection revealed that the number of bacteria in cells infected with the WT strain was much higher than in cells infected with the *ΔSopB* strain (**Figure 5D**). This may explain the two phenotypes of *S*. Infantis causing varying degrees of pyroptosis: the WT strain has a greater bacterial load and stimulates the inflammasome more strongly.

### WT *S*. Infantis Causes More Severe Intestinal Inflammatory Damage Than the *ΔSopB* Strain

**Figure 6:**
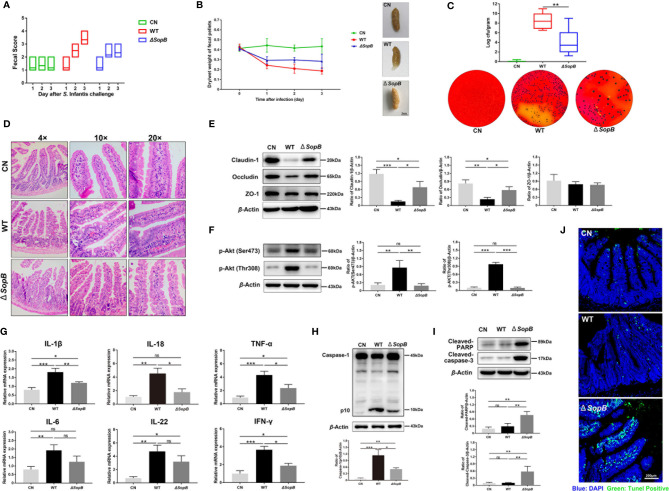
WT *S*. Infantis caused more severe intestinal inflammatory damage to the C57BL/6 mice gut than the Δ*SopB* mutant. **(A)** The fecal score curve of mice in three groups after *Salmonella* infection. The fecal status of mice was scored daily. **(B)** Results of the dry/wet weight ratio of feces. The starting day of the experiment was considered as day 0 (24 h after streptomycin gavage). Three representative feces images of each group are shown on the right side of the curve. **(C)** Quantification of fecal bacterial burdens on day 3. Three representative plots of bacterial colonies images in each group are below the curve. **(D)** Ileum representative photomicrographs of sections stained with H&E. **(E, F)** Western blot analysis of Claudin-1, Occludin, p-Akt (Ser473), and p-Akt (Thr308) protein levels of three groups. **(G)** Expression of IL-1β, IL-18, IL-6, IL-22, TNF-α, and IFN-γ mRNA in ileal tissues. **(H, I)** Western blot analysis of Caspase-1, Cleaved-caspase-3, and Cleaved-PARP protein levels of three groups. **(J)** The representative images of TUNEL fluorescence staining in ileum tissue pathological sections. Blue: DAPI; Green: TUNEL positive cells. Data were presented as mean ± SEM. (n = 6). Data were presented as the mean ± SEM from three independent experiments (n = 3). *P < 0.05, **P < 0.01, ***P < 0.001. CN, control. ns, no significant difference.

In order to verify our findings *in vivo*, we infected the C57BL/6 mouse model with *Salmonella*. The severity of diarrhea was determined by fecal score and the dry/wet weight of fecal pellets. The results revealed that WT *S*. Infantis caused more severe diarrhea than the *ΔSopB* strain (**Figures 6A, B**). In addition, the fecal bacterial load in the WT group was also significantly higher than the *ΔSopB* group (**Figure 6C**). Since *Salmonella* infection can cause severe ileal injury, the pathological changes in the ileum were examined. Infection with WT *S*. Infantis resulted in more severe ileal damage than infection with the *ΔSopB* strain (**Figures 6D, E**). Consistent with the *in vitro* results, p-Akt (Ser473 and Thr308) was found to be highly expressed in the WT group (**Figure 6F**). As shown in **Figure 6G**, infection with WT *S*. Infantis significantly increased the mRNA level of inflammatory factors, while the mRNA level of inflammatory factors induced by the *ΔSopB* strain was lower compared to WT *S*. Infantis. In combination with the detection of caspase-1 level (**Figure 6H**), we discovered that the infection with WT strain led to more severe intestinal inflammatory injury. Furthermore, immunoblotting and the TUNEL fluorescence assay showed that the *ΔSopB* strain caused more severe apoptosis of intestinal cells than the WT stain (**Figures 6I, J**). Intestinal cells infected with the *ΔSopB* strain may shed rapidly from the epithelium through apoptosis and be eliminated from the body, reducing the gut *Salmonella* load and inflammatory response.

In conclusion, *S*. Infantis delayed the death of infected Caco-2 cells through intermittent activation of Akt mediated by *SopB*, allowing intracellular cytosolic bacteria sufficient time for replication and resulting in more severe intestinal inflammation.

## Discussion

Many pathogenic bacteria that are closely related to public health reproduce intracellularly, enhancing their virulence. Invasion and colonization in epithelial cells are crucial processes in *Salmonella* pathogenesis (36). The replication of cytosolic *Salmonella* is key to the early establishment of the infection (10, 11). In this study, we elucidated the mechanism by which cytosolic *S*. Infantis delayed the death of infected epithelial cells *via* intermittent Akt phosphorylation mediated by *SopB*.

*Salmonella* utilizes *SopB* to activate Akt, suggesting that it plays an important role in regulating host cell survival (18, 19). However, the distribution of *SopB*-dependent Akt phosphorylation in epithelial cells remained unclear. A single-cell approach was used to evaluate the relationship between *SopB* and Akt phosphorylation. Because *SopB* was transported to cells to play a role in mediating both actin-dependent and myosin II-dependent bacterial invasion, we attributed the induction of the first wave of Akt phosphorylation to its residual activity. The second wave of Akt phosphorylation activity only occurred at 3–4 hpi, with Akt phosphorylation only strongly induced in infected cells containing hyper-replicating cytosolic bacteria. Notably, there was no Akt phosphorylation at any other time in all infected cells. We hypothesized that the second wave of Akt activation was due to the residual SPI-1 activity of bacteria escaping from the SCV. This was similar to Akt phosphorylation by *S*. Typhimurium: the first stage was widely induced during invasion, and the second stage was induced only in the infected cells containing cytosolic *Salmonella* (20). However, there were some differences between the two types of *Salmonella*: the first phase of Akt phosphorylation induced by *S*. Typhimurium was largely depleted by 3 hpi, whereas the first phase of Akt phosphorylation induced by *S*. Infantis induced was almost depleted at 1 hpi. The second stage of Akt activation induced by *S*. Infantis induction was occurred at 3–4 hpi, while *S*. Typhimurium-induced Akt activation was later at 6 hpi (20). Previous studies have shown that *S*. Infantis was less invasive and induced considerably weaker enteritis than *S*. Typhimurium (37). These differences were attributed to the low expression of SPI-1 in *S.* Infantis than that in *S*. Typhimurium (37).

*SopB* delayed the inevitable and rapid apoptosis of intestinal epithelial cells. For *Salmonella*, the most obvious advantage gained by inhibiting apoptosis is time, which allows *Salmonella* to establish an intracellular stronghold to rapidly proliferate, as well as regulate its and the host’s gene expression to prepare for the spread of infection in the gut. The mitochondrion is the primary organelle for endogenous apoptosis (30). In this study, we discovered that cytosolic *S*. Infantis phosphorylated Akt through *SopB* to (i) reposition Bcl-2 to regulate the permeability of the mitochondrial outer membrane by inhibiting Bax translocation from cytosol to the mitochondria; (ii) maintain mitochondrial dynamic network homeostasis; (iii) phosphorylate Bad and Caspase-9 to inhibit apoptosis of infected cells, thereby maintaining the mitochondrial membrane and network homeostasis to suppress the apoptosis of infected cells.

Importantly, we discovered that *SopB*-mediated Akt phosphorylation delayed the pyroptosis of infected cells. Recently, it has been reported that *SopB* inhibited the activation of NLRC4 inflammasome in BMDMs through an Akt signal-dependent process (35). Another study found that *SopB* promoted YAP phosphorylation through Akt in B cells, thereby inhibiting the assembly of the inflammasome (38). The activation of the inflammasome is a crucial step in inducing of pyroptosis. Our findings demonstrated that *SopB*-mediated Akt phosphorylation also delayed the activation of caspase-1 and pyroptosis in Caco-2 cells. Compared to apoptosis, pyroptosis occurs faster and is accompanied by the release of pro-inflammatory factors, such as IL-1β and IL-18 (21–23). Inflammatory factors increase the number of inflammatory cells and aggravate the inflammatory response, which is a vital defense mechanism for the host to combat pathogenic microorganism infection (39). Although the inflammatory response accelerates the elimination of pathogens, it also alters the intestinal environment by disrupting gut microbiota and causing a burst of intestinal electron acceptors (40, 41). An alteration in the intestinal ecosystem provides variables for pathogens to gain a growth advantage to spread infection. In this study, *S*. Infantis rapidly induced the pyroptosis within 2 h after Akt phosphorylation diminished. The WT *S*. Infantis caused more severe intestinal inflammation *in vivo* than the *SopB* mutant (**Figure 6**). We hypothesized that *SopB* contributes to the rapid proliferation of bacteria in the intestinal infection phase of *S*. Infantis, which leads to inflammatory response, intestinal damage, and other intestinal environmental variables, as well as provides favorable conditions for the subsequent establishment of long-term colonization infection in the gut.

In conclusion, this study demonstrated that the *S*. Infantis SPI-1 effector *SopB* acts as a pro-survival factor in epithelial cells by inhibiting apoptosis and delaying pyroptosis. *S*. Infantis gained sufficient time to proliferate through the regulation of host cell death, which resulted in inflammation and suitable conditions for the spread and colonization of pathogens in the gut. As it is interesting that bacterial pathogens can manipulate host cell death mechanisms to enhance pathogen survival and spread infection, further studies focusing on how *S*. Infantis regulates different types of host cell death to cause enteritis will be performed. This study also provides a solid theoretical basis as well as potential drug targets for the treatment of salmonellosis.

## Data Availability Statement

The raw data supporting the conclusions of this article will be made available by the authors, without undue reservation.

## Ethics Statement

The animal study was reviewed and approved by the Guidelines for Laboratory Animal Use and Care from the Chinese Center for Disease Control and Prevention and the Rules for Medical Laboratory Animals (1998) from the Chinese Ministry of Health, under the approval of the Animal Ethics Committee of the China Agricultural University.

## Author Contributions

B-XC designed and performed the experiments. Y-NL, N-L, and Y-HZ participated and assisted in the experiments as well as provided advice during the procedure. L-XY and S-YC contributed to data analysis. B-XC drafted the manuscript, and J-FW critically revised the manuscript. J-FW was responsible for obtaining funding and overseeing the project. All authors contributed to the article and approved the submitted version.

## Funding

This work was supported by the National Key R&D Program of China (Project No. 2017YFD0502200).

## Conflict of Interest

The authors declare that the research was conducted in the absence of any commercial or financial relationships that could be construed as a potential conflict of interest.

## Publisher’s Note

All claims expressed in this article are solely those of the authors and do not necessarily represent those of their affiliated organizations, or those of the publisher, the editors and the reviewers. Any product that may be evaluated in this article, or claim that may be made by its manufacturer, is not guaranteed or endorsed by the publisher.
